# Genetic mapping of time-to-maturity trait in *hypogaea* x *fastigiata* peanut background reveals a significant effect of pod-related and flowering pattern

**DOI:** 10.1186/s12870-025-07707-z

**Published:** 2025-12-01

**Authors:** Srinivas Kunta, Naga Sravani Gogisetty, Gilad Ben-Israel, Yael Levy, William Mlelwa, Nevet Zur Biton, Ye Chu, Peggy Ozias-Akins, Ran Hovav

**Affiliations:** 1 Department of Field Crops, Institute of Plant Sciences, Agriculture Research Organization - The Volcani Center, HaMakkabbim Road, POB 15159, Rishon LeZiyyon, 7505101 Israel; 2 Faculty of Agricultural, Food and the Environmental Quality Sciences, The Hebrew University of Jerusalem, POB 12, Rehovot, 7610001 Israel; 3 Department of Horticulture, Institute of Plant Breeding, Genetics and Genomics, University of Georgia, Tifton, GA 31793 USA

**Keywords:** Peanut (*Arachis hypogaea*), Time to maturity, Fastigiata, Hypogaea, Genetic linkage map

## Abstract

**Background:**

Domesticated peanut (*Arachis hypogaea* L.) comprises two main subspecies, ssp. *fastigiata* and ssp. *hypogaea*, which differ in several characteristics, most notably time-to-maturation (TTM). Despite TTM‘s siginicance for adaptability, yield and quality, its genetic control in peanut remains largely unknown. Here, a recombinant inbred line (RIL) population, derived from a *hypogaea* (late-maturing) X *fastigiata* (early-maturing) cross, was used to dissect the genetics of TTM across three environments and to determine the associations with other traits such as plant architecture and pod/seed-related traits. A high-density genetic map that comprises 4671 SNP markers was used.

**Results:**

Eighty-one quantitative trait loci (QTLs) were identified for all traits. Six loci were found for TTM, four of which (on A02, A05, A07 and A10) were stable and consistent across all three environments. Most TTM QTLs had small-to-medium effects except one QTL, *qMIA07*, which explained up to 14.3% PVE. Gene Onthology analyses showed that *qMIA07* is enriched in processes that connected to pod/seed size. Indeed, *qMIA07* and other three TTM QTLs were co-localized with pod/seed-related QTLs like pod/seed weight, number of double/multiple-seeded pods and number pods per plant. Flowering pattern, which is considered a TTM-affecting trait, was also co-localized with TTM on LG B02 but in only one environment. However, analysis of this QTL in a Near-Isogenic background confirmed a significant effect for flowering patern on TTM.

**Conclusions:**

This study demonstrates that seed/pod traits and flowering pattern are important factors that should be considered in introgressing early maturation from *fastigiata* background into *hypogaea* germplasm.

**Supplementary Information:**

The online version contains supplementary material available at 10.1186/s12870-025-07707-z.

## Background

Peanut (*Arachis hypogaea* L.) is one of the most important legume and oilseed crops, widely cultivated in tropical and subtropical regions. Global peanut seed production is estimated at 55.66 million tons (USDA FAS reports, August 2024). Two main subspecies (ssp.) of peanut are grown worldwide: *A. hypogaea* ssp. *fastigiata* and *A. hypogaea* ssp. *hypogaea*. *Fastigiata* accounts for > 90% of global peanut production and includes the ‘Spanish’ and ‘Valencia’ marketing types. Spanish peanuts have a higher oil content than other types making them ideal for oil production. They are also used for direct consumption and peanut butter production in certain regions. Valencia peanuts, grown on a smaller scale, are primarily used for the boiled peanut market. Subspecies *hypogaea* includes the ‘Virginia’ and ‘Runner’ market types, which produce larger seeds and pods than *fastigiata*. Virginia peanuts are commonly used for salting, confections, and in shell roasting. Runner peanuts, known for their excellent flavor, roasting characteristics, and high yields, have largely replaced Spanish types in the USA and other regiones. They are widely used in peanut butter production and as salted nuts.

Subspecies *fastigiata* and *hypogaea* exhibit distinct morphological and agronomic traits. As noted earlier, the Virginia and Runner market types of ssp. *hypogaea* produce larger seeds than the Spanish and Valencia types of ssp. *fastigiata*. Another key difference lies in their growth habit. *Fastigiata* types have erect lateral branches, as reflected in their scientific name, whereas *hypogaea* types display spreading or bunch laterals [1]. Flowering pattern (FP), also termed “branching pattern”, is another distinguishing characteristic. *Fastigiata* types bear flowers on the mainstem and follow a sequential flowering pattern, while *hypogaea* types lack flowers on the mainstem and exhibit an alternate flowering pattern [1].

Another key agronomic difference between *ssp. fastigiata* and *ssp. hypogaea* is their growing period, or time to maturity (TTM), which is crucial for crop adaptability and yield. *Fastigiata* varieties have a shorten growing period, typically maturing in 90–120 days, whereas *hypogaea* varieties take longer, maturing in 130–170 days [2]. As a result, *fastigiata* cultivars are well-suited for regions with a short rainy season or two growing cycles per year, such as India, West Africa, Southeast Asia, and Central America. In contrast, *hypogaea* cultivars thrive in areas with long summers and access to supplemental irrigation, such as the USA and the Middle East. In general, early-maturing *fastigiata* varieties tend to have lower yields than late-maturing varieties due to their shorter growing season. However, some early-maturing varieties can achieve high pod yields due to their rapid growth and high harvest index [3].

Due to climate change and increasing water scarcity, peanut breeding efforts have focused on developing early-maturing varieties while maintaining high yields and favorable agronomic traits. However, achieving this balance presents significant challenges. One major limitation is the underground development of peanut pods, which makes assessing maturity difficult. The current standart for evaluating maturity, the hull-scrape method [4], is labor-intensive and somewhat subjective. Additionally, peanut maturity is quantitative trait with low to medium heritability [5–8] and is influenceed by multiple genes and environmental factors [9, 10]. Further complicating breeding efferts is the peanut’s narrow genetic base, which stems from an evolutionary bottleneck coused by a single hybridization event followed by polyploidization. This genetic constraint, along with the difficulty of crossing between the wild diploid species and domesticated tetraploid species, poses additional hurdels [11]– [12]. Moreover, the widespread reliance on a small number of elite varieties in breeding programs has further restricted genetic diversity, limiting the availablity of molecular markers for breeding assistance.

Utilizing *fastigiata* germplasm to introduce early maturation into *hypogaea* cultivars appears to be a promising strategy. However, the genetics of TTM in *fastigiata* × *hypogaea* background remains largely unexplored. Limited studies have attempted to identify genomic loci controlling crop maturity using low-density genetic maps derived from *fastigiata* ×*hypogaea* breeding materials [13–15], resulting in the identification of QTLs with small-to-medium effects. For example, a QTL for pod maturity (PM45) was reported in a Runner × Spanish cross using SSR markers on LG5, explaining 17.7% of the phenotypic variance [13]. Using an amphidiploid (A. ipaensis × A. duranensis) × Spanish background, QTLs associated with pod maturity (PMAT) were mapped on LGs B03, B06, and B11, each explaining 9.3–12.6% of the variation [15]. Additional studies in Spanish × Virginia and Spanish × Spanish populations identified maturity-related loci (PM01, PM02) with moderate effects on pod number and maturity [16]. Other investigations identified QTLs for sound mature kernel percentage (SMK%) in Spanish × Spanish populations, detecting multiple loci across AhV, AhVI, AhIX, AhXXI, AhI, and AhVIII linkage groups, with relatively small effects ranging from 3.3% to 7.41% [17].

Other distinguishing traits between *fastigiata* and *hypogaea* may influence the efficiency of selection for TTM. For instance, flowering pattern impacts the plant’s reproductive-to-vegetative ratio and has been hypothesized as a key factor affecting maturity [2]. Additionally, seed and pod size have been reported to correlate negatively with maturity rate in Virginia X Spanish peanut crosses [6, 18]. Conversely, early maturity has been linked to easily measurable traits, such as pod and seed size as well as plant architecture. These correlations could facilitate indirect selection for early maturation in cases where early these traits do not constitute a major breeding objective [5].

The present study was initiated to investigate the quantitative genetic control of peanut maturity through direct trait measurement and its indirect relationships with other traits, such as flowering pattern, growth habit and pod/seed characteristics. To achieve this, a recombinant inbed lines (RIL) population derived from a *hypogaea* × *fastigiata* cross was used. High-density genetic mapping (HDGM) was performed using a modified version of an existing genetic map [19] and field phenotyping across three environments. The analysis identified consistent QTLs associated with TTM and their correlations with other traits, providing valuble tools for improving selection efficiency and accelerating genetic gains in the development of early-maturing peanut cultivars.

## Results

### Phenotypic evaluation of MI and the other traits

**Fig. 1:**
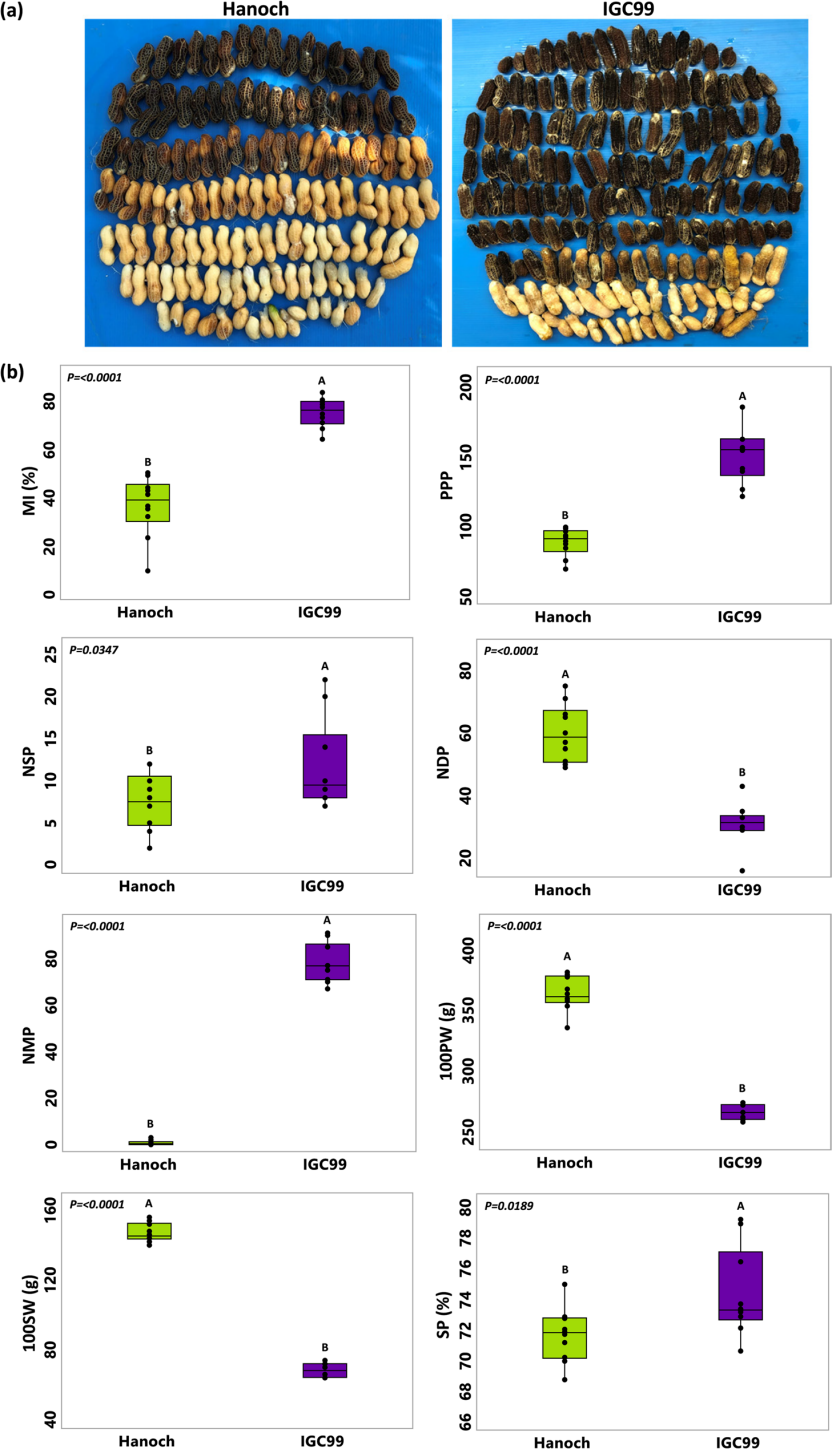
Phenotypic characterization of MI and additional traits in ‘Hanoch’ and ‘IGC99’. **a** Example of MI morphology based on mesocarp color, derived from pressure-washed exocarps of fresh pods harvested from ‘Hanoch’ and ‘IGC99’. **b** Comparisons of MI, PPP, NSP, NDP, NMP, 100PW, 100SW, and SP between ‘Hanoch’ and ‘IGC99′. Data represent the mean from three growth environments (n = 10). MI, maturity index (%); PPP, pods per plant; NSP, number of single-seeded pods; NDP, number of double-seeded pods; NMP, number of multiple-seeded pods; 100PW (g), 100 pod weight; 100SW (g), 100 seed weight; SP (%), shelling percentage

The visual differences in maturity level between the parental lines, Hanoch and IGC99, are shown in Fig. 1a. The parental MI values were collected from the three field experiments, and their overall mean data are presented in Fig. 1b. A highly significant difference was observed between the parental lines for MI, with values of 36.6 ± 12.4 and 75.3 ± 5.9 for Hanoch and IGC99, respectively (P = < 0.0001). Additionally, significant differences were found between the parental lines for all other measured component traits, including PPP, NDP, NMP, 100PW, 100SW, NSP and SP (Fig. 1b).

**Fig. 2:**
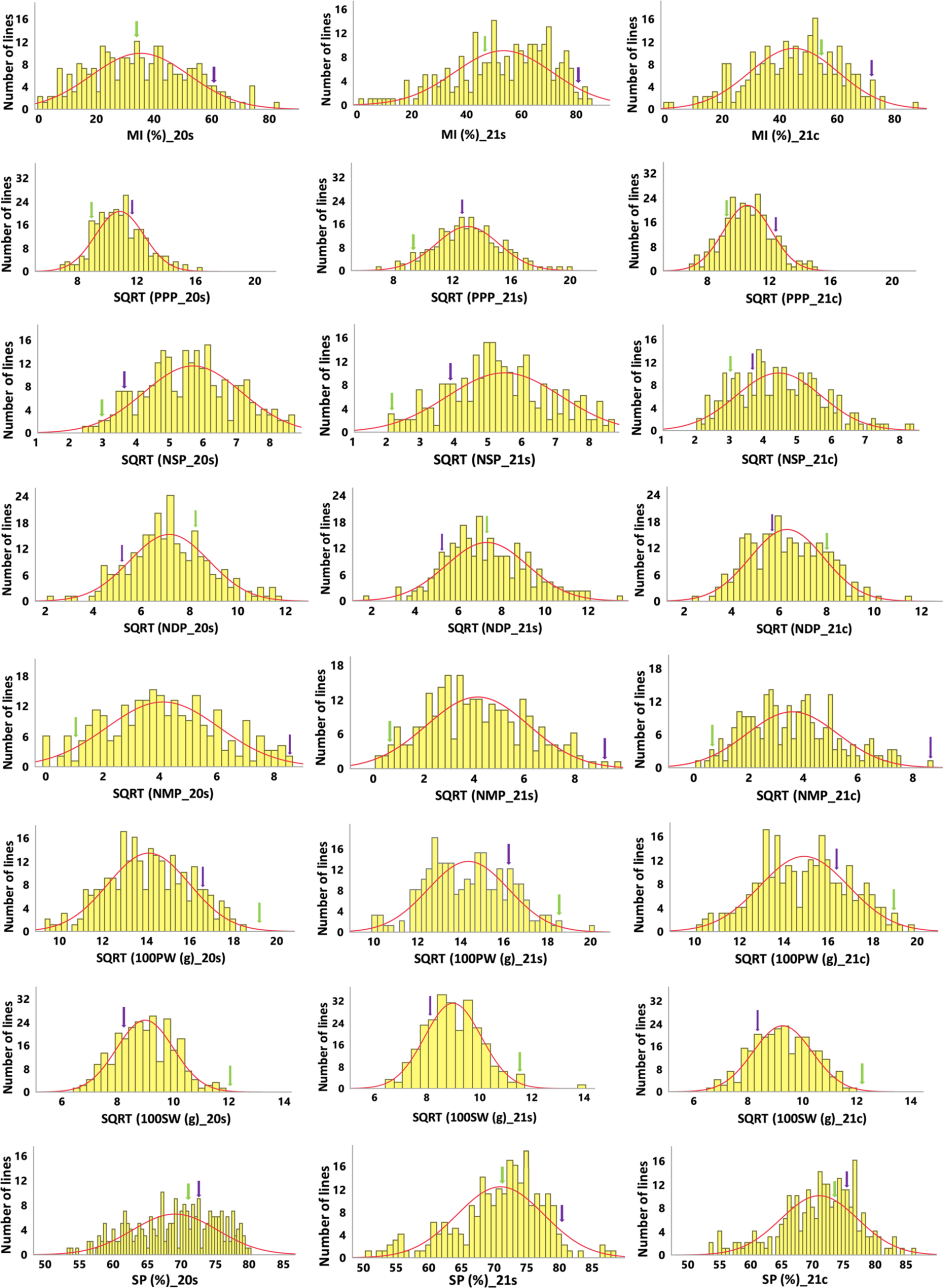
Phenotypic distribution of MI and other traits in 2020 s (left panel), 2021 s (middle panel) and 2021c (right panel). The X-axis corresponds to the phenotypic trait values based on the average of three replicates from each envrionment and the Y-axis corresponds to the number of RIL lines. Arrows indicate the phenotypic values for ‘Hanoch’ (green) and ‘IGC99’ (purple). A normal distribution curve is indicated by the red line. MI, maturity index (%); PPP, pods per plant; NSP, number of single-seeded pods; NDP, number of double-seeded pods; NMP, number of multiple-seeded pods; 100PW (g), 100 pod weight; 100SW (g), 100 seed weight; SP (%), shelling percentage. _20s, season 2020 south; _21s, season 2021 south; _21c, season 2021 central. SQRT, square root transformed values

Most of the RIL population distributions showed significant deviations from normality (Table 1). Therefore, a square root transformation was applied to most traits (Fig. 2). After transformation, many traits displayed a near-normal distribution (Fig. 2; Table S1), except for NMP in all environments, MI and NSP in two environments, and NDP at 21c. The range of RIL values for MI extended significantly beyond the mean of the late-maturing parental line)Hanoch(, indicating skewness towards late maturation in this population. Transgressive segregation was also observed in most traits, except for NSP, NMP 100PW and 100SW. ANOVA tests revealed a significant effect for blocks, RIL, environment, and RIL X environment interaction across all studied traits (Table 2). As a result, genetic mapping was conducted separately for each environment. The broad-sense heritability (H^2^) for MI was estimated at 0.67, while heritability estimates for other traits ranged from 0.38 (PPP) to 0.71 (100SW) (Table 2).

**Table 1:** Summary statistics of MI and the other traits among parents and RILs

		Parents			RILs			
	Variables	Han	IGC99	Student t-test	Mean ± SD	Min	Max	Sig. of S-W test ^i^
2020s	MI (%)^a^	31.4	61.5	0.0004^*^	34.6 ± 16.9	0	81.1	0.0324^**^
	PPP^b^	87	135	0.0001^*^	116.2 ± 33.8	47	213.3	0.0752 ^j^
	NSP^c^	8.3	12.3	0.2336^ns^	32.7 ± 15.4	5.7	74.3	0.0542 ^j^
	NDP ^d^	64	31.3	< 0.0001^*^	52.8 ± 24.3	4.5	137.7	0.1676 ^j^
	NMP ^e^	1	77.5	< 0.0001^*^	20.6 ± 17.2	0	72.7	0.0279^**j^
	100PW (g) ^f^	371.7	265.3	< 0.0001^*^	200.1 ± 52.7	87	337	0.226 ^j^
	100SW (g) ^g^	149.7	68.3	< 0.0001^*^	80.6 ± 18.4	43.3	136	0.2338 ^j^
	SP (%) ^h^	70.9	72.1	0.0366^**^	68.8 ± 6.3	53.3	79.9	< 0.0001^*^
2021s	MI (%)	46.4	80.7	0.0004^*^	52.7 ± 17.7	0	83.8	< 0.0001^*^
	PPP	89.3	165	< 0.0001^*^	167.9 ± 51.3	47.1	297.7	0.9073 ^j^
	NSP	4.5	9.5	0.0194^**^	30.1 ± 16.1	4.1	72.4	0.0007^*j^
	NDP	50.5	29.5	0.0011^*^	54.7 ± 27.4	2.7	138.9	0.0425^**j^
	NMP	0.5	83.5	0.0061^*^	20.2 ± 17.8	0	90.7	0.0083^*j^
	100PW (g)	348	263	0.0205^**^	207.1 ± 53.8	97	394.2	0.0463^**j^
	100SW (g)	141	65	0.0003^*^	80.3 ± 18.8	41.7	134.1	0.0052^*j^
	SP (%)	71.5	79	0.0272^**^	70.7 ± 6.7	50.1	88.3	< 0.0001^*^
2021c	MI (%)	55.6	73.6	0.0046^*^	44.3 ± 15.5	44.3	86.3	0.1692
	PPP	88.7	147.5	0.0013^*^	112.1 ± 33.4	46.5	222.2	0.3371
	NSP	8.5	14	0.0670^ns^	20.9 ± 11.8	4.2	69.2	0.0027^*j^
	NDP	63.5	30.5	0.0041^*^	41.6 ± 20.2	5.9	128.1	0.0521 ^j^
	NMP	0.5	78	0.0081^*^	15.2 ± 13.3	0	73.4	0.0012^*j^
	100PW (g)	357	273	0.0014^*^	223.7 ± 59.2	100	381.2	0.1061 ^j^
	100SW (g)	145	71	0.0004^*^	85.8 ± 20.1	44	137.6	0.0772 ^j^
	SP (%)	73.9	75.1	0.5738^ns^	70.8 ± 6.2	53.3	85.9	< 0.0001^*^

^a^ MI (%), maturity index;^b^ PPP, pods per plant; ^c^ NSP, number of single-seeded pods; ^d^ NDP, number of double-seeded pods; ^e^ NMP, number of multiple-seeded pods; ^f^ 100PW (g), 100 pod weight; ^g^ 100SW (g), 100 seed weight; ^h^ SP (%), shelling percentage; ^i^ significance for normality test by Shapiro - Wilk test; ^j^ normality test by Shapiro - Wilk test on square root transformed values; ^*^,^**^ and ^ns^ mean significant at *P* < 0.01, *P* < 0.05 and not significant, respectively. *Han* Hanoch

**Table 2:** Analysis of variance and heritability for MI and the component traits for the Hanoch X IGC99 RIL population across three environments. Block [Environment] indicates the nested effect of the blocks within each environment

Trait	Variables	DF	Mean square	F Ratio	*P*-Value	H^2 i^
MI ^a^	Block [Environment]	6	8917.04	123.01	< 0.0001^*^	0.67
	Environment	2	69940.82	964.83	< 0.0001^*^	
	RIL	293	2243.60	30.95	< 0.0001^*^	
	RIL x Environment	573	212.79	2.93	< 0.0001^*^	
	Error	1729	72.49			
PPP ^b^	Block [Environment]	6	13024.77	14.16	< 0.0001^*^	0.38
	Environment	2	790102.56	859.2	< 0.0001^*^	
	RIL	293	9542.87	10.37	< 0.0001^*^	
	RIL x Environment	568	2777.33	3.02	< 0.0001^*^	
	Error	1715	919.57			
NSP ^c^	Block [Environment]	6	6349.13	48.97	< 0.0001^*^	0.50
	Environment	2	32979.18	254.39	< 0.0001^*^	
	RIL	293	1435.61	11.07	< 0.0001^*^	
	RIL x Environment	573	187.06	1.44	< 0.0001^*^	
	Error	1649	129.63			
NDP ^d^	Block [Environment]	6	7587.91	33.2	< 0.0001^*^	0.59
	Environment	2	43432.16	190.03	< 0.0001^*^	
	RIL	293	4253.96	18.61	< 0.0001^*^	
	RIL x Environment	584	497.01	2.17	< 0.0001^*^	
	Error	1670	228.54			
NMP ^e^	Block [Environment]	6	691.22	10.33	< 0.0001^*^	0.70
	Environment	2	7409.57	110.78	< 0.0001^*^	
	RIL	293	2033.18	30.4	< 0.0001^*^	
	RIL x Environment	585	143.23	2.14	< 0.0001^*^	
	Error	1672	66.88			
100PW ^f^	Block [Environment]	6	8869.38	14.9	< 0.0001^*^	0.68
	Environment	2	107189.94	180.17	< 0.0001^*^	
	RIL	292	21940.91	36.88	< 0.0001^*^	
	RIL x Environment	584	2159.04	3.62	< 0.0001^*^	
	Error	1663	594.92			
100SW ^g^	Block [Environment]	6	712.58	12.24	< 0.0001^*^	0.71
	Environment	2	7794.17	133.94	< 0.0001^*^	
	RIL	293	2723.64	46.8	< 0.0001^*^	
	RIL x Environment	585	246.41	4.23	< 0.0001^*^	
	Error	1669	58.19			
SP ^h^	Block [Environment]	6	245.66	17.27	< 0.0001^*^	0.68
	Environment	2	1208.45	84.99	< 0.0001^*^	
	RIL	292	363.24	25.54	< 0.0001^*^	
	RIL x Environment	576	27.69	1.94	< 0.0001^*^	
	Error	1651	14.21			

^a^ MI, maturity index; ^b^ PPP, pods per plant; ^c^ NSP, number of single-seeded pods; ^d^ NDP, number of double-seeded pods; ^e^ NMP, number of multiple-seeded pods; ^f^ 100PW (g), 100 pod weight; ^g^ 100SW (g), 100 seed weight; ^h^ SP (%), shelling percentage; ^i^ Broad sense heritability; ^*^ significant at *P* < 0.01; ^ns^ non-significant. ANOVA was performed in QTL IciMapping software

Segregation analysis of the maturity index revealed that models assuming multiple major genes provided a substantially better fit than those based on polygenes alone. Among the 24 tested models (Table S2), the four-major-gene model with three epistatic genes and one additive gene (4MG-EEEA) exhibited the lowest AIC average value (6457.7) and passed all goodness-of-fit tests (U¹, U², U³, nW, and Dn; all *p* > 0.95). This model explained the largest proportion of variance, with major-gene variance estimated at ~ 310 and broad-sense heritability attributable to major genes reaching 92.8%. Alternative models with similar structure, such as 4MG-EEA and 4MG-CEA, also showed strong support (AIC < 6460; H² >91%), but provided slightly inferior fit compared with the 4MG-EEEA model. Collectively, these results demonstrate that maturity index in this population is predominantly controlled by a small number of major genes with both additive and epistatic effects, consistent with a discrete segregation pattern rather than a purely polygenic architecture.

**Fig. 3:**
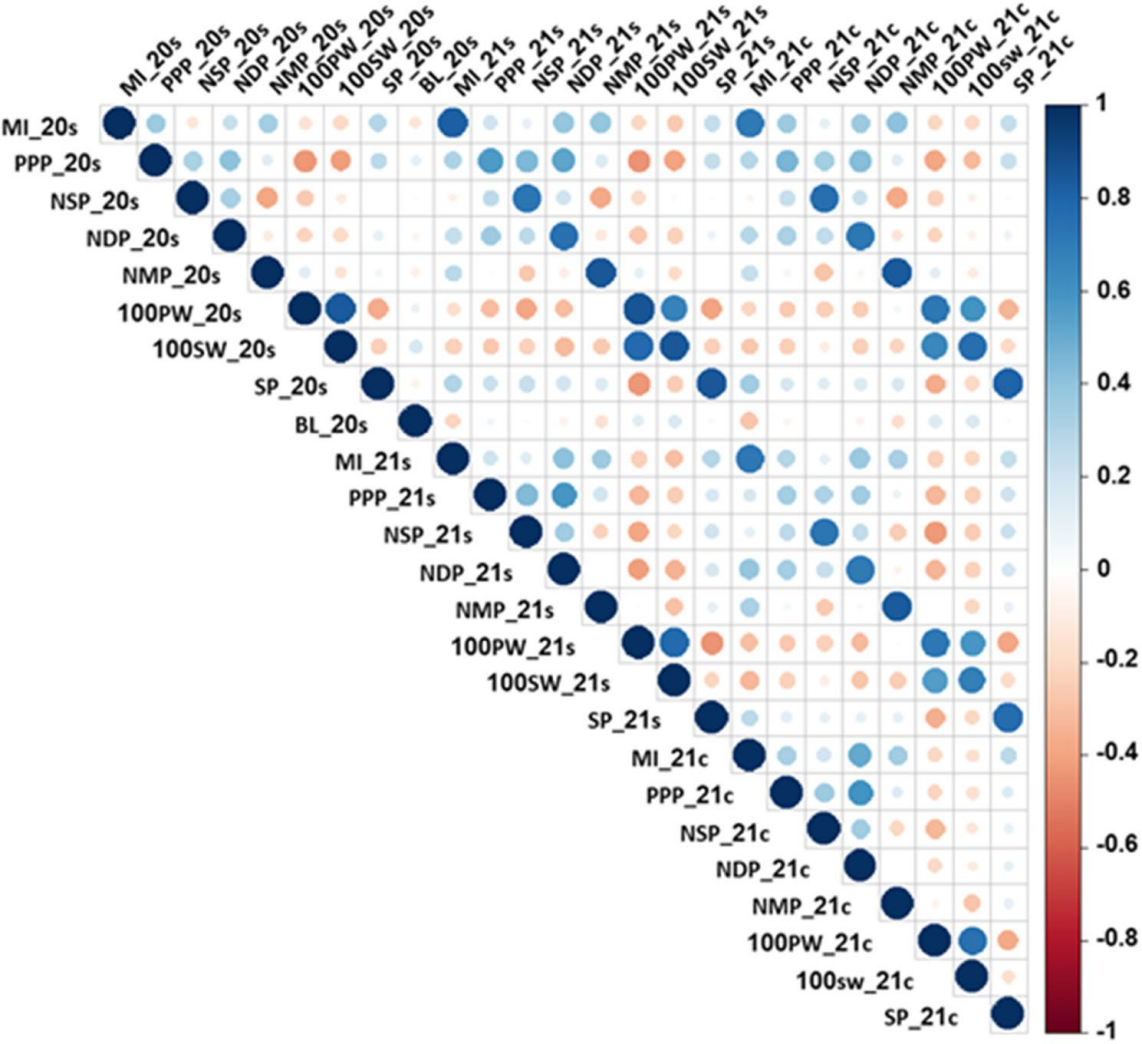
Pearson correlations for MI and the component traits in the Hanoch X IGC99 RIL population across three environments. Colors represent the correlation coefficient (r) ranging from positive (blue) to negative (red). Circle size indicates significance level, with larger and darker-colored dots representing significant correlations at *p* < 0.05. Negative correlations are denoted by (-), while values without circles indicate non-significant correlations at *p* > 0.05. MI, maturity index; PPP, pods per plant; NSP, number of single-seeded pods; NDP, number of double-seeded pods; NMP, number of multiple-seeded pods; 100PW (g), 100 pod weight; 100SW (g), 100 seed weight; SP (%), shelling percentage; BL, branch length. 2020 s; environment 2020 south; 2021 s; environment 2021 south, and 2021c; environment 2021 center

Pearson correlations among the measured traits were calculated for each environment (Fig. 3). The correlation between the three environments for MI was 0.81 (*P* < 0.0001) between 2020 s and 2021 s, 0.71 (*P* < 0.0001) between 2020 s and 2021c and 0.72 (*P* < 0.0001) between 2021 s and 2021c, with an average of 0.74. These results suggest a relatively higher genetic heritability for MI than estimated by the ANOVA. The maturity index (MI) showed a highly significant positive correlation with PPP, NDP, NMP and SP, and a negative correlation with 100PW and 100SW across all three environments. In addition, MI in 2020s/2021s environments was positively correlated with NSP in 2021 s and 2021c, while MI in 2021s/2021c was negatively correlated with BL in 2020s. Significant correlations were also observed among other traits. PPP was positively correlated with NSP and NDP, and negatively correlated with 100PW, 100SW and partially positively with NMP; NSP positively with NDP, negatively with NMP, 100PW, 100SW; and NDP negatively with 100PW and 100SW. NMP showed a significant negative correlation with 100SW. SP showed a significant positive correlation with PPP, negative with 100PW and 100SW, and a partial positive correlation with NSP, NDP and NMP (Fig. 3).

The effects of growth habit (BH), flowering pattern (FP) and branching rate (BR) on the MI trait were analyzed using a t-test (Fig. S1). A significant but small effect of BH, FP and BR on MI was observed across the three environments. Among BH phenotypes, erect-type lines exibited higher MI values than spreading or bunch-type lines. For FP, MSF-plus lines showed higher MI values than MSF-minus lines. Similarly, in BR, lines with ‘up to 5 laterals’ had higher MI values than those with ‘many laterals’.

### Genetic linkage map and trait mapping

MI and the other traits were mapped by using a high-density genetic map previously reported by Kunta et al. [19], with slight modifications due to the RIL population size. The modified genetic map was constructed using 6871 input SNPs and 254 RILs. The resulting linkage groups covered 2793 cM and contained 4671 markers, which were assigned to 21 LGs (Fig. S2; Table S3). Linkage groups were assigned to the respective pseudomolecules (chromosomes) of *A. hypogaea* cv. Tifrunner reference genome, as described [19]. The 4671 loci spanned a total physical distance of 2237.69 Mbp with an average physical interval of 0.53 Mbp between loci (Fig. S2; Table S3). The average recombination rate was 1.12 cM/Mbp.

**Fig. 4:**
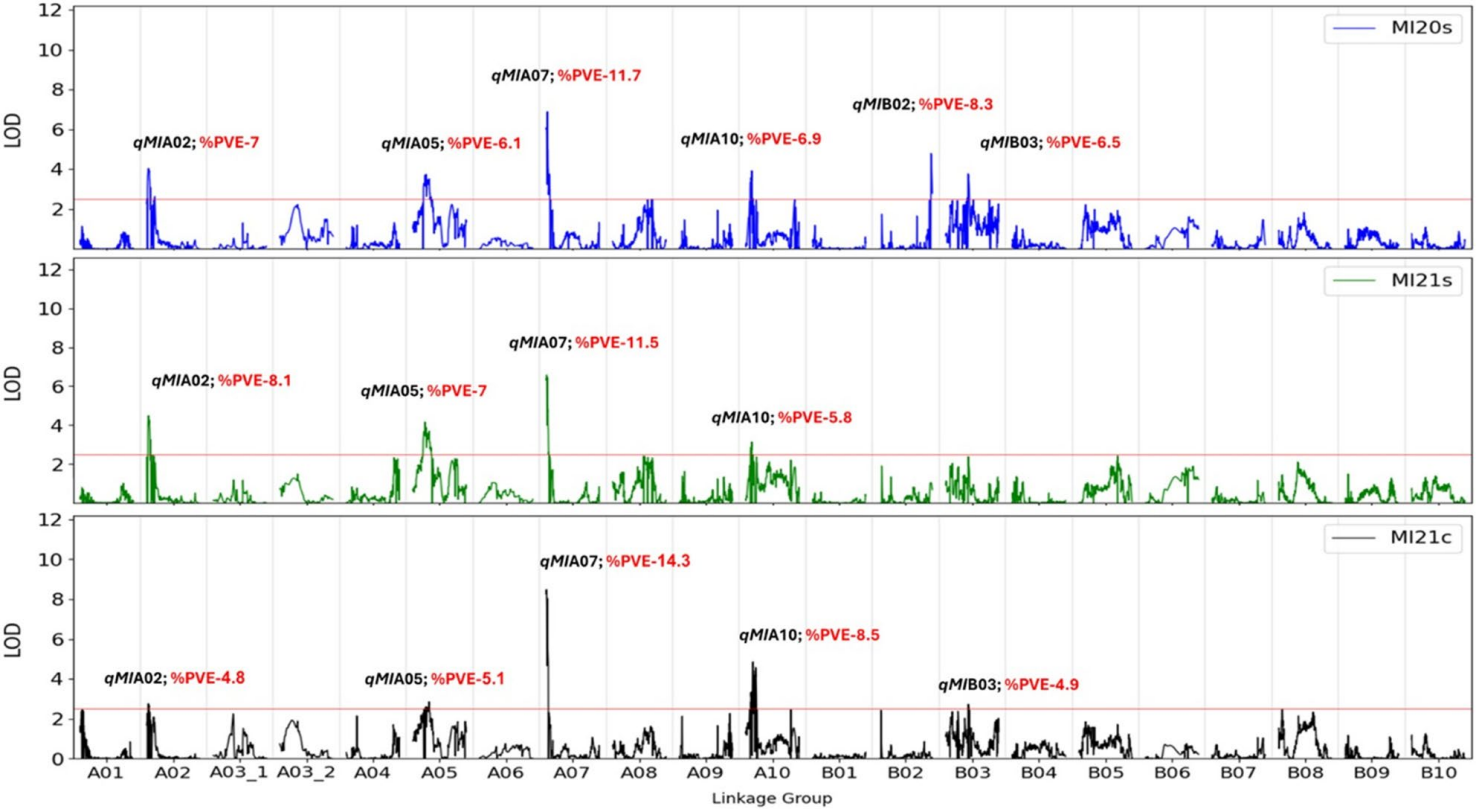
Whole-genome QTL analysis for the MI trait among the Hanoch × Harari RIL population in three environments. % PVE, percentage of phenotypic variance explained. %PVE was derived from the marker with the highest value within each QTL. The red line defines the threshold LOD score of 2.5

A total of 15 QTLs were identified for MI trait on six LGs, explaining 4.8 to 14.3% PVE (Fig. 4; Table 3). Out of these, four consistent QTLs were found across the three environments, one less consistent QTL was significant in only two environments and the last one in a single environment (Fig. 4; Table 3). One major and consitant QTL *qMIA07a*,-*b*,-*c* was observed on LG A07 between AX-176794752_A07 - AX-147227012_A07, spanning over 1.7 Mbp, with PVE values of 11.7, 11.5 and 14.3% for 2020 s, 2021 s and 2021c, respectively. The next consistent QTL *qMIA02a*,-*b*,-*c* was observed on LG A02 within marker interval of AX-147240517_A02 - AX-176804252_A02, spanning 1.4 Mbp, explaining 7, 8.1, and 4.8% PVE across three environments. The other two consistent QTLs across three environments, *qMIA05a*,*-b*,*-c* (AX-176796538_A05 - AX-176793478_A05; 2.8 Mbp; 6.1, 7, 5.1% PVE, respectively) and *qMIA10a*,*-b*,*-c* (AX-177640375_A10 - AX-176815985_A10; 3.2 Mbp; 6.9, 5.8, 8.5% PVE, respectively) were identified on LG A05 and A10, respectively. Out of the four consistent QTLs, in two, the allele conferring early maturation was derived from IGC99 (*qMIA05a*,-*b*,-*c* and *qMIA10a*,-*b*,*-c*) and in the other two from Hanoch (*qMIA02a*,-*b*,*-c* and *qMIA07a*,-*b*,-*c*). QTL *qMIB03a*,-*b*, with flanking markers AX-176806036_B03 - AX-176823583_B03, were identified on LG B03, explaining 6.5 and 4.9% of the phenotypic variation, respectively. The early maturation conferring allele of *qMIB03a*,-*b* was derived from IGC99. The last MI QTL, *qMIB02a*, located between *AhTFL1* indel - AX-176803063_B02, spanning 1.1 Mbp, explaining 8.3% PVE, was identified on LG B02. IGC99 was the parent that conferred the early maturation allele of *qMIB02a*. The *qMIB02* locus co-localized with *qMSFB02* and *qBRB02* on LG B02 over 3.1 Mbp between AX-176791582_B02 - AX-176803063_B02 interval, a locus that was previously showed to be containing an indel of *AhTFL1* gene [19].

For the pod-related/seed-related traits, a total of 60 QTLs were found, out of which 17 major QTLs (6 NMP, 4 100PW, 7 100SW) were identified across all three environments with 4.9–18.4% PVE (Fig. S2**;** Table 3). Interestingly, four out of six loci for MI shared common QTL regions with pod/seed-related traits QTLs. *qMIA02a*,-*b*,-*c* and *qNDPA02a*,-*b*,-*c* overlapped over 3.1 Mbp between the marker interval AX-147240517_A02 - AX-147213196_A02 on LG A02. *qMIA05a*,-*b*,-*c* and *qNMPA05.2a*,-*b*,-*c* shared a common region of 10.5 Mbp between AX-176796538_A05 - AX-176810047_A05 on LG A05. Interestingly, the major QTL for MI, *qMIA07a*,*-b*,*-c*, was co-localized with *qPPPA07a*,-*c*, *qNDPA07a*,-b,-*c*,* q100PWA07a*,-*b*,-*c*, and *q100SWA07a*,-*b*,-*c* between AX-176794752_A07 - AX-147227012_A07 (1.7 Mbp) on LG A07. Maturity index QTL *qMIB02* and *qPPPB02* shared 1.1 Mbp common QTL region between *AhTFL1* indel - AX-176803063_B02 marker interval on LG B02. Other pod/seed-related QTLs that were not co-localized with MI included QTLs *qNDPA09a*,-*b*,-*c* that overlapped with *qNMPA09a*,-*b*,-*c* and *qNSPA09.2* between AX-147234119_A09 - AX-176819108_A09 over 3.6 Mbp on LG A09, and QTLs *q100PWB08a*,-*b*,-*c* co-localized with *q100SWB08a*,-*b*,-*c* and *qSPB08a*,-*b*,-*c* over 1.6 Mbp between AX-177640947_B08 - AX-147257475_B08 marker interval on LG B08 (Fig. S2**;** Table 3).

**Table 3:** QTLs identified for MI and the other traits in the Hanoch X IGC99 RIL population across three environments

Trait	Year	QTL	LG^a^	Position (cM)	Flanking Markers^d^	Peak marker^d^	Physical position range (Mbp)	LOD	PVE (%)^b^	ADD^c^	Parent ADD effect
MI	2020s 2021s	*qMIA02a* *qMIA02b*	A02	6.69	AX-147240517_A02 - AX-147213196_A02	AX-176795792_A02	5.2–8.3	4 4.47	7 8.1	−4.62275 −5.21314	Hanoch
MI	2020s	*qMIA05a*	A05	20.962	AX-176804489_A05 - AX-176810468_A05	AX-176811362_A05	27.3–37.8	3.46	6.1	4.29006	IGC99
MI	2020s	*qMIA07a*	A07	3.373	AX-176794752_A07 - AX-147254562_A07	AX-147226951_A07	0.1–2.9	6.88	11.7	−5.92579	Hanoch
MI	2020s	*qMIA10a*	A10	24.37	AX-147235016_A10 - AX-176820298_A10	AX-176815491_A10	2.3–3.03	3.92	6.9	4.48779	IGC99
MI	2020s	*qMIB02*	B02	163.972	*AhTFL1* indel - AX-176803063_B02	*AhTFL1* indel	119.1–120.2	4.79	8.3	4.89464	IGC99
MI	2020s	*qMIB03a*	B03	74.897	AX-176806036_B03 - AX-176823583_B03	AX-176795824_B03	20.1–24.3	3.68	6.5	4.41519	IGC99
MI	2021s	*qMIA05b*	A05	20.962	AX-176796538_A05 - AX-176810047_A05	AX-176811362_A05	26.7–37.8	3.84	7	4.81991	IGC99
MI	2021s	*qMIA07b*	A07	3.373	AX-176794752_A07 - AX-147254502_A07	AX-147226951_A07	0.1–2.4	6.46	11.5	−6.12353	Hanoch
MI	2021s	*qMIA10b*	A10	24.37	AX-177640375_A10 - AX-176815492_A10	AX-176815491_A10	2.3–2.5	3.14	5.8	4.2964	IGC99
MI	2021c	*qMIA02c*	A02	6.69	AX-147240517_A02 - AX-176804252_A02	AX-176795792_A02	5.2–6.6	2.68	4.8	−3.48177	Hanoch
MI	2021c	*qMIA05c*	A05	26.941	AX-176806538_A05 - AX-176793478_A05	AX-176820250_A05	29.7–32.5	2.85	5.1	3.55958	IGC99
MI	2021c	*qMIA07c*	A07	0.102	AX-176794752_A07 - AX-147227012_A07	AX-176795541_A07	0.1–1.8	8.47	14.3	−5.99666	Hanoch
MI	2021c	*qMIA10c*	A10	30.65	AX-177640375_A10 - AX-176815985_A10	AX-177639716_A10	2.3–5.5	4.85	8.5	4.54052	IGC99
MI	2021c	*qMIB03b*	B03	74.897	AX-176791349_B03 - AX-176813244_B03	AX-176795824_B03	22.6–23.1	2.73	4.9	3.49248	IGC99
PPP	2020s	*qPPPA07a*	A07	0.102	AX-176794752_A07 - AX-147227012_A07	AX-176795541_A07	0.1–1.8	4.94	8.6	−10.894	Hanoch
PPP	2021s	*qPPPA07b*	A07	0.102	AX-176794752_A07 - AX-176795541_A07	AX-176819550_A07	0.1–0.1	2.9	5.3	−13.281	Hanoch
PPP	2021s	*qPPPA01*	A01	0.726	AX-147238153_A01 - AX-176824158_A01	AX-176818026_A01	0.05–0.06	2.73	5	12.743	IGC99
PPP	2021c	*qPPPA07c*	A07	0.204	AX-176794752_A07 - AX-147226949_A07	AX-147226951_A07	0.1–1.3	2.79	4.9	−7.57054	Hanoch
PPP	2021c	*qPPPB02*	B02	163.972	*AhTFL1* indel - AX-176803063_B02	*AhTFL1* indel	119.1–120.2	5.15	8.9	9.96972	IGC99
PPP	2021c	*qPPPB03*	B03	19.486	AX-176800555_B03 - AX-147215424_B03	AX-147243171_B03	3.2–4.05	3.02	5.3	7.92976	IGC99
NSP	2020s	*qNSPA09.1a*	A09	94.256	AX-177640143_A09 - AX-177639683_A09	AX-176816808_A09	103.7–105.9	3.45	6.1	−4.35686	Hanoch
NSP	2020s	*qNSPA09.2*	A09	115.254	AX-147234119_A09 - AX-177641266_A09	AX-177642648_A09	110.8–115.9	4.91	8.5	−5.11577	Hanoch
NSP	2020s	*qNSPB10*	B10	108.555	AX-176822190_B10 - AX-176820249_B10	AX-176809030_B10	139.09–141.5	3.1	5.5	4.108	IGC99
NSP	2021s	*qNSPA07*	A07	0.102	AX-176794752_A07 - AX-176819896_A07	AX-176819550_A07	0.1–0.2	3.01	5.3	−4.7666	Hanoch
NSP	2021s	*qNSPA09b*	A09	93.741	AX-147233827_A09 - AX-147233874_A09	AX-176815953_A09	104.2–105.4	2.75	4.9	−4.5206	Hanoch
NSP	2021s	*qNSPB04a*	B04	81.795	AX-147247750_B04 - AX-147247896_B04	AX-176820656_B04	84.3–109.8	2.86	5.1	4.57154	IGC99
NSP	2021c	*qNSPA01*	A01	114.098	AX-176810167_A01 - AX-176795944_A01	AX-176822520_A01	97.5–98.5	3.11	5.5	2.81734	IGC99
NSP	2021c	*qNSPA09c*	A09	94.46	AX-147233783_A09 - AX-177639606_A09	AX-147233874_A09	102.9–106.	3.52	6.2	−2.97235	Hanoch
NSP	2021c	*qNSPB04b*	B04	73.577	AX-176823074_B04 - AX-176817221_B04	AX-176802636_B04	18.7–107.6	3.36	5.9	2.88851	IGC99
NDP	2020s 2021s 2021c	*qNDPA02a* *qNDPA02b* *qNDPA02c*	A02	7 7.128 7	AX-147240517_A02 - AX-147213196_A02	AX-176822526_A02	5.2–8.3	4.65 5.69 4.21	8.1 9.8 7.4	−7.101 −9.31522 −5.6318	Hanoch
NDP	2020s	*qNDPA03_2*	A03_2	20.139	AX-147218061_A03 - AX-176801936_A03	AX-176791844_A03	135.7–136.3	3.16	5.6	5.80174	IGC99
NDP	2020s	*qNDPA07a*	A07	3.373	AX-176794752_A07 - AX-176807692_A07	AX-147226951_A07	0.1–1.3	3.21	5.7	−5.90104	Hanoch
NDP	2020s	*qNDPA09a*	A09	108.95	AX-176797227_A09 - AX-176819108_A09	AX-177643598_A09	104.4–114.4	5.25	9.1	−7.42638	Hanoch
NDP	2020s	*qNDPB02*	B02	33.265	AX-176805995_B02 - AX-176792812_B02	AX-147213125_B02	7.1–10.2	3.16	5.6	−5.89792	Hanoch
NDP	2021s	*qNDPA07b*	A07	3.373	AX-176794752_A07 - AX-147227012_A07	AX-147226951_A07	0.1–1.8	5.18	9	−8.87288	Hanoch
NDP	2021s	*qNDPA09b*	A09	108.95	AX-147234119_A09 - AX-147234396_A09	AX-177643598_A09	110.8–114.1	3.54	6.2	−7.33654	Hanoch
NDP	2021s	*qNDPB03a*	B03	117.522	AX-147217633_B03 - AX-176805534_B03	AX-176813062_B03	130.08–132.01	3.21	5.7	7.02834	IGC99
NDP	2021c	*qNDPA07c*	A07	3.373	AX-176794752_A07 - AX-147226949_A07	AX-147226951_A07	0.1–1.3	2.92	5.1	−4.68046	Hanoch
NDP	2021c	*qNDPA09c*	A09	108.95	AX-147234119_A09 - AX-176819108_A09	AX-177643598_A09	110.8–114.4	4.77	8.3	−5.89775	Hanoch
NDP	2021c	*qNDPB03.1b*	B03	117.522	AX-176800235_B03 - AX-176805534_B03	AX-176813062_B03	129.9–132.4	3.38	5.9	5.01261	IGC99
NDP	2021c	*qNDPB03.2*	B03	138.215	AX-176805993_B03 - AX-176791678_B03	AX-176801586_B03	135.7–138.4	2.95	5.2	4.64667	IGC99
NMP	2020s	*qNMPA05.1a*	A05	1.234	AX-176807363_A05 - AX-176824027_A05	AX-176822590_A05	6.4–10.6	5.83	10	5.48682	IGC99
NMP	2020s	*qNMPA05.2a*	A05	43.189	AX-176793806_A05 - AX-176820120_A05	AX-177638594_A05	12.07–97.01	11.24	18.4	7.46845	IGC99
NMP	2020s	*qNMPA09a*	A09	113.002	AX-177642426_A09 - AX-177641266_A09	AX-177644154_A09	110.2–115.9	5.77	9.9	5.4545	IGC99
NMP	2021s	*qNMPA05b*	A05	42.371	AX-176807363_A05 - AX-176801332_A05	AX-176807899_A05	6.4–98.6	9.97	16.5	7.87209	IGC99
NMP	2021s	*qNMPA09b*	A09	109.052	AX-147234119_A09 - AX-176811256_A09	AX-147234289_A09	110.8–116.4	6.01	10.3	6.10355	IGC99
NMP	2021c	*qNMPA04*	A04	48.396	AX-147255195_A04 - AX-147247065_A04	AX-176797560_A04	5.4–9.3	2.85	5	3.05859	IGC99
NMP	2021c	*qNMPA05c*	A05	42.985	AX-176807363_A05 - AX-176809797_A05	AX-176822023_A05	6.4–92.9	8.18	13.8	5.00891	IGC99
NMP	2021c	*qNMPA09c*	A09	109.052	AX-177642426_A09 - AX-177637151_A09	AX-147234289_A09	110.2–117.1	6.35	10.9	4.44055	IGC99
100PW	2020s	*q100PWA07a*	A07	3.168	AX-176794752_A07 - AX-147227012_A07	AX-177639855_A07	0.1–1.8	5.37	9.3	16.3819	IGC99
100PW	2020s	*q100PWB08a*	B08	38.335	AX-177643024_B08 - AX-177644281_B08	AX-177640759_B08	4.04–119.5	9.59	16	−21.4315	Hanoch
100PW	2021s	*q100PWA07b*	A07	3.168	AX-176794752_A07 - AX-147254502_A07	AX-177639855_A07	0.1–2.4	6.27	10.8	18.0151	IGC99
100PW	2021s	*q100PWB08b*	B08	38.335	AX-177643633_B08 - AX-177644281_B08	AX-177640759_B08	3.4–119.5	10.39	17.2	−22.7261	Hanoch
100PW	2021c	*q100PWA07c*	A07	0.102	AX-176794752_A07 - AX-147227003_A07	AX-176795541_A07	0.1–1.3	3.8	6.7	15.6437	IGC99
100PW	2021c	*q100PWB08c*	B08	38.335	AX-176815348_B08 - AX-147257745_B08	AX-177640759_B08	4.01–15.3	8.1	13.6	−22.2217	Hanoch
100SW	2020s 2021s	*q100SWA05a* *q100SWA05b*	A05	99.117	AX-147250275_A05 - AX-176801364_A05	AX-147223559_A05	106.9–108.7	6.42 5.87	11 10.1	−6.22278 −6.46318	Hanoch
100SW	2020s	*q100SWA07a*	A07	3.168	AX-176794752_A07 - AX-147227012_A07	AX-177639855_A07	0.1–1.8	5.94	10.2	5.98929	IGC99
100SW	2020s	*q100SWB08a*	B08	32.245	AX-177643024_B08 - AX-177643976_B08	AX-147257433_B08	4.04–11.3	9.15	15.3	−7.33792	Hanoch
100SW	2020s	*q100SWB08.1a*	B08	80.27	AX-176799643_B08 - AX-177640257_B08	AX-177638150_B08	26.01–122.5	4.87	8.5	−5.45946	Hanoch
100SW	2021s	*q100SWA07b*	A07	3.168	AX-176794752_A07 - AX-147254502_A07	AX-177639855_A07	0.1–2.4	5.28	9.1	6.14055	IGC99
100SW	2021s	*q100SWB08b*	B08	32.245	AX-177643024_B08 - AX-177641417_B08	AX-147257433_B08	4.04–8.1	8.28	13.9	−7.59364	Hanoch
100SW	2021s	*q100SWB08.1b*	B08	80.27	AX-176807875_B08 - AX-176823271_B08	AX-177638150_B08	30.4–119.2	3.68	6.5	−5.17214	Hanoch
100SW	2021c	*q100SWA05c*	A05	99.117	AX-176821239_A05 - AX-176794905_A05	AX-147223559_A05	95. 6–109.8	6.6	11.3	−6.8687	Hanoch
100SW	2021c	*q100SWA07c*	A07	0.204	AX-176794752_A07 - AX-147226949_A07	AX-176795541_A07	0.1–1.3	2.91	5.1	4.65645	IGC99
100SW	2021c	*q100SWB08c*	B08	32.245	AX-147257294_B08 - AX-177641417_B08	AX-147257433_B08	4.5–8.1	6.58	11.2	−6.85716	Hanoch
SP	2020s	*qSPB06*	B06	24.137	AX-147251710_B06 - AX-147251757_B06	AX-147260021_B06	2.4–6.3	3.32	5.9	1.73496	IGC99
SP	2020s	*qSPB08a*	B08	30.388	AX-177640947_B08 - AX-147257475_B08	AX-177641788_B08	5.6–7.2	3.65	6.4	1.8106	IGC99
SP	2020s	*qSPB10a*	B10	96.411	AX-177637327_B10 - AX-147264929_B10	AX-177640478_B10	137.07–138.01	4.24	7.4	−1.93172	Hanoch
SP	2021s	*qSPB08b*	B08	38.335	AX-177644216_B08 - AX-177639173_B08	AX-177640759_B08	4.5–7.4	5.24	9.1	2.27797	IGC99
SP	2021c	*qSPB08c*	B08	38.335	AX-147257294_B08 - AX-177641372_B08	AX-177640759_B08	4.5–24.9	5.37	9.3	1.98045	IGC99
SP	2021c	*qSPB10b*	B10	69.411	AX-177637327_B10 - AX-177643957_B10	AX-177640479_B10	137.07–139.6	4.6	8	−1.8247	Hanoch
BL	2020s	*qBLB05*	B05	120.912	AX-147250415_B05 - AX-176802394_B05	AX-147251194_B05	136.9–159.3	11.82	19.3	−5.16861	Hanoch
BL	2020s	*qBLB09*	B09	6.57	AX-147223990_B09 - AX-147232169_B09	AX-177644036_B09	0.6–2.03	4.59	8	3.33539	IGC99
TESTA	2020s	*qTCA03_1*	A03_1	93.786	AX-176820223_A03 - AX-176810993_A03	AX-147216730_A03	30.7–42.5	6.06	10.7	−0.143549	Hanoch
TESTA	2020s	*qTCA03_2*	A03_2	1.142	AX-176800578_A03 - AX-147245610_A03	AX-176818479_A03	131.9–133.9	5.21	9.3	−0.133682	Hanoch
TESTA	2020s	*qTCB10*	B10	32.273	AX-176820176_B10 - AX-176815494_B10	AX-177643724_B10	1.9–10.3	3	5.4	0.101756	IGC99
PH	2020s	*qPHA05*	A05	99.117	AX-147250275_A05 - AX-147223560_A05	AX-147223559_A05	106.9–107.5	3.5	6.4	−0.124825	Hanoch

^a^LG, linkage group; ^b^ PVE, Phenotypic variance explained; ^c^ ADD, additive effect; ^d^ Affymetrix Axiom sequence ID. *MI* maturity index, *PPP* pods per plant, *NSP* number of single-seeded pods; *NDP* number of double-seeded pods, *NMP* number of multiple-seeded pods, 100PW (g), 100 pod weight; 100SW (g), 100 seed weight; *SP (%)* shelling percentage, *BL* branch length, *TESTA* testa color, *PH* pod hardness

For BL, two QTLs were identified in 20 s, explaining 8 and 19.3% PVE, respectively (Table 3). The QTL *qBLB05* co-localized with the BH QTL, *qBHB05*, spanning approximately 9 Mbp between the marker interval AX-176798604_B05 - AX-176802394_B05 on LG B05 (Fig. S2). For PH, a single QTL, *qPHA05* was identified on LG A05, within the marker interval AX-147250275_A05 to AX-147223560_A05, spanning 0.6 Mbp and explaining 6.4%PVE. Since minimal differences were observed in the PH phenotype among the same RILs across 20s, 21 s and 21c, the same locus, *qPHA05*, was denoted for all three environments (Table 3). For TC, three QTLs were identified. A major QTL, *qTCA03_1* (on LG A03_1) and a minor QTL, *qTCA03_2* (on LG A03_2) were identified on LGs A03_1 and A03_2 explaining 10.7 and 9.3% PVE, respectively. The other QTL, *qTCB10*, was found on LG B10, explaining 5.4% PVE. The TC phenotype remained consistent across all three environments, leading to the use of the same values for the RILs in subsequent analyses (Table 3).

### Functional annotation of the *qSSB02* QTL region

To identify genes and genetic pathways potentially associated with MI, 127 genes located within *MIA07* (A7166054-A71842128) QTL region were extracted from the Tifrunner reference genome annotation and analyzed against the entire gene set in the genome. Gene Ontology (GO) annotation showed that the majority of enriched genes had specific functional assignments: in the biological processes category, terms included regulation of response to stress, cysteine, cellulose, glucan and carbohydrates metabolic processes; In the molecular function category, terms included ion binding, deacetylase activity, carbohydrate symporter activity and cellulose synthase activity; and in the cellular component category terms included mitochondrial ribosome and phosphopyruvate hydratase complex (Fig. S3).

### Validation of the *qMIB02* effect in Hanoch genetic background

**Fig. 5:**
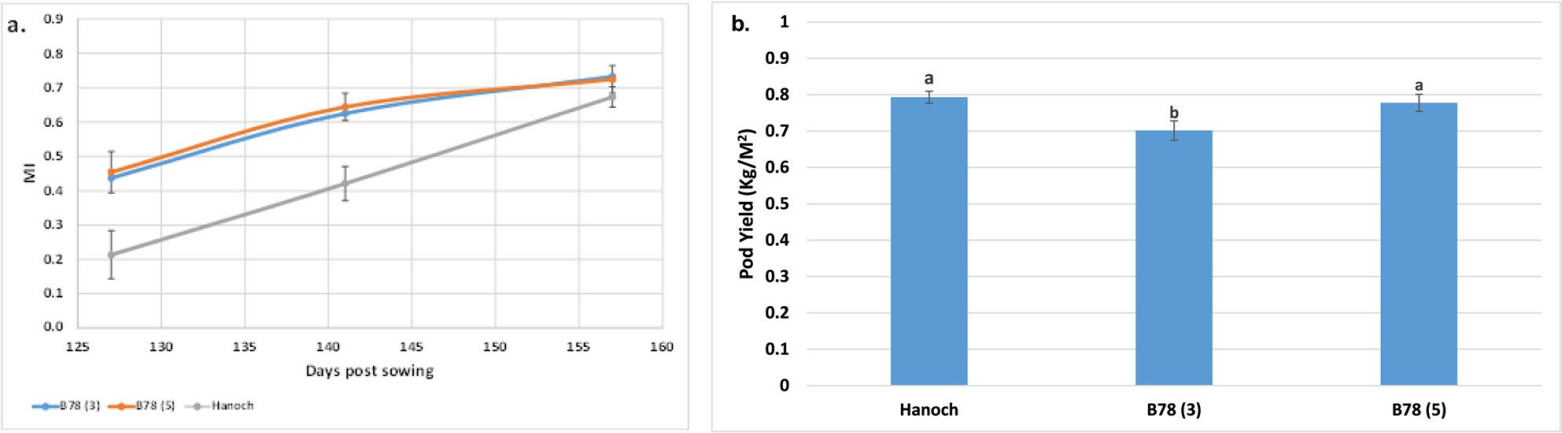
**a**. Maturity index (MI) of Hanoch vs. B78 grown on three rows per bed (B78(3)) and B78 grown on five rows per bed (B78(5)), in three different growing time points. **b**. Total pod yield (Kg/M^2^) of Hanoch vs. B78(3) and B78(5). Groups with different letters (**a**,**b**) are significantly different at p<0.05

Although *qMIB02* was statistically significant in only one environment (20 S), its effect was further investigated within the genetic background of the late-maturing parental line, Hanoch. This investigation was driven by previous studies indicating that flowering pattern may directly influence maturity level under certain genetic background [2, 17]. The maturity progression curves of the varieties Hanoch and a Hanoch-based line B78 (near isogenic line with sequential flowering pattern) throughout the growing season are presented in Fig. 5a. A significant difference in maturity level between the two varieties was observed as early as the first assessment date (127 days from sowing), with Hanoch exhibiting a maturity level of approximately 20% compared to about 42% in line B78. This difference decreased over the growing period but remained statistically significant at the final assessment date. Notably, line B78 was ready for harvest at 145 days from sowing, approximately two weeks earlier than Hanoch. No differences in maturity level were found between B78 grown at three rows per bed and B78 grown at five rows per bed. Pod yield results are presented in Fig. 5b. A significant difference in yield was found between Hanoch and B78 grown in three rows per bed. However, increasing the planting density of B78 to five rows per bed nearly compensated for this yield difference.

## Discussion

Improving early maturing, high-yielding varieties has been a long term goal for peanut breeders. The rapid rise in global population, climate change, and the shrinking availability of arable land and freshwater resources underscore the need for developing early-maturing, high-yield peanut varieties. Early maturity enhances adaptation to shortened growing seasons [20], whereas late maturation is associated with prolonged pod-filling and increased yield potential [21].

Crop maturation is influenced by genetic factors and their interactions with the environment. In legumes, TTM is primarily regulted by two developmental aspects, flowering time and inflorescence architecture [22–27]. However, peanut TTM exhibits a distinct genetic architecture compared to other legumes. Although peanut is classified as a short-day plant, earlier studies have shown that its flowering time is minimally affected by photoperiod and has only a minor effect on TTM [28]. Additionaly, the classical definition of inflorescence architecture determinate vs. indeterminate growth - does not fully apply to peanut TTM. This is due to the presence of strong indeterminate lateral shoot tips in both wild and domesticated peanut species [29]. Therefore, alternative models are required to accurately describe the genetic regulation of TTM in peanut.

In our previous studies, RIL populations derived from ssp. *hypogaea* crosses (Virginia × Virginia, Runner × Virginia) were used to investigate the genetic control of TTM in peanut [7, 8]. Through this work, four consistent QTLs were identified with medium-to-low effects were identified, and it was suggested that harvest index plays a key role in TTM within ssp. *hypogaea*. However, no specific trait was found to directly influence the harvest index, leaving the exact mechanism for early maturation unclear. These early studies were limited by low phenotypic variation within the gerplasm and a relatively small number of genetic markers used for mapping.

The current study expands on this research by introducing ssp. *fastigiata* as a new source for TTM analysis, providing both wider phenotypic and genotypic variation compared to ssp. *hypogaea*. The maturity index among *hypogaea* x *fastigiata* RILs displayed significant variation (ranging from 0 to 86) with higher heritability estimations (0.67) than previously reported in peanut ([5–8], highlighing a strong genetic basis for TTM in this background. Additionally, this study incorporated a higher-density genetic map (HDGM), analyzing ~ 14.3% of polymorphic markers on the SNP array. The resulting HDGM contained 21 LGs with 4671 markers, achieving an average map density of 0.7 cM per locus, covering over 90% of the peanut reference genome. Other recent HDGM in peanut include 2808 markers spanning 1308.2 cM [30], 2996 SNPs and 330 SSRs covering 1822.83 cM [31], 3630 SNPs spanning 2098.14 cM [32], 5120 SNPs covering 3179 cM [33], and 8869 SNPs (whole genome population re-sequencing) with a map length of 3120 cM [34]. Depite the low polymorphism levels observed in peanut, recent advances in SNP-based high-throughput sequencing technologies [35–41] and the availability of the tetraploid reference genome [42] have significantly enhanced genetic studies compared to previous efforts that relied on SSR markers with very low sequence variation [43–45].

By integrating high-density genetic mapping with multi-environment field trials, we identified six quantitative trait loci (QTLs) associated with TTM. Notably, four of these QTLs (*qMIA02*,* qMIA05*,* qMIA07*,* and qMIA10*) were consistently detected across all three environments, demonstrating their stability and potential utility in peanut breeding programs. The detection of four stable QTLs for maturity index provides strong evidence for discrete genetic control of this trait. Notably, the segregation analysis independently supported this conclusion by identifying four major genes, suggesting that the QTLs likely reflect the effects of these underlying loci. The strongest QTL, qMIA07, explained up to 14.3% of the phenotypic variance (PVE) and exhibited enrichment for gene ontology (GO) categories related to pod and seed size, indicating a possible mechanistic link between these traits and TTM. The identified TTM QTLs from the current HDGM are novel compared to those previously reported (Table 4).

**Table 4:** List of maturity related QTLs reported in the previous studies in peanut

Trait name	QTL ^a^ name	Cross type	Type of markers	LG ^b^	Peak marker/Marker interval	Physical position (Tetraploid)	PVE ^c^ (*R*^2^)	Reference
Pod maturity	*Mature %*	Runner × Spanish	SSR	5	PM45	-	17.7	[13]
Number of mature pods/plant	*PM01*	Spanish × Virginia Spanish × Spanish Spanish × Spanish	SSR^*^	4	pPGSseq17E3 - EM-87	-	11.9	[16]
	*PM02*			7	pPGPseq3E10 - GA131	-	12.3	
Pods maturity	*PMAT* ^WW^	Spanish × Amphidiploid AiAd (*A. ipaensis* KG30076 × *A.duranensis* V14167)^x4^	SSR	B03	PM003_B	-	9.3	[15]
	*PMAT* ^WL^			B06	TC19F05_B	-	9.6	
	*PMAT* ^WL^			B11	TC2A02_B	-	12.6	
Sound mature kernel percentage (SMK%)	*qSMK_WW09B* ^WW^	Spanish × Spanish	SSR	AhV	GM633-TC2D08	-	4.41	[17]
	*qSMK_WW09B* ^WW^			AhVI	Seq9H08a-IPAHM171	-	3.5	
	*qSMK_WW09B* ^WW^			AhIX	Seq2B09-TC5A06	-	5.1	
	*qSMK_WW09B* ^WW^			AhXXI	Seq19D09-TC7E04	-	7.41	
	*qSMK_WS09B* ^WS^			AhI	GM635b-GM635a	-	3.3	
	*qSMK_WS09B* ^WS^			AhVIII	TC9F10-TC6H03	-	3.85	
Maturity index (%)	*qMIA04a*	Virginia × Virginia	SNP	A04	AX-176802283_A04 - AX-176815499_A04	117.6–125.6.6.6	9.9	[7]
	*qMIA04b*			A04	AX-176819644_A04 - AX-176815499_A04	118.6–125.6.6.6	11.9	
	*qMIB03a*			B03	AX-176807311_B03 - AX-176806413_B03	2.8–4.6	9.3	
	*qMIB03b*			B03	AX-176807311_B03 - AX-176801237_B03	2.8–5.7	9.9	
	*qMIB05_2*			B05_2	AX-147251167_B05 - AX-176821336_B05	156.5–158.9.5.9	10.2	
	*qMIB06*			B06	AX-147252043_B06 - AX-176807746_B06	11.3–16.6	9.8	
Maturity index (%)	*qMIA04a*	Virginia × Runner	SNP	A04	AX-176819644_A04 - AX-147221341_A04	118.7–126.6.7.6	11.5	[8]
	*qMIA04b*			A04	AX-176819644_A04 - AX-147221341_A04	118.7–126.6.7.6	8.1	
	*qMIA08_2a*			A08_2	AX-177637914_A08 - AX176821868_A08	48.4–51.4	7.3	
	*qMIA08_2b*			A08_2	AX-177639781_A08 - AX-176821672_A08	50.3–51.3	8.2	
	*qMIB02*			B02	AX-176794798_B02 - AX-176812478_B02	105.8–105.9.8.9	9	
	*qMIB04*			B04	AX-176802465_B04 - AX-176799466_B04	56.4–57.1	6.8	

^a^ QTL, quantitative trait loci; ^b^ LG, linkage group; ^c^ PVE, phenotypic variance explained (%); ^*^ integrated linkage map; ^WW^ well-watered; ^WL^ water-limited; ^WS^ water stress

The most notable outcome of this study was the connection between MI and pod/seed-related traits. MI was positively correlated with PPP, NDP, NMP and SP, and negatively correlated with 100PW and 100SW across all three environments. These findings align with previous studies that have suggested a genetic association between seed/pod traits and TTM in peanut [5, 13, 15]. Specifically, the co-localization of TTM QTLs with pod/seed-related QTLs (such as those governing pod weight, seed weight, and pod number per plant) suggests that genetic factors regulating pod development and yield components may also influence TTM. This relationship is particularly evident at the qMIA07 locus, which overlaps with multiple pod-related QTLs like *qPPPA07a*,* b*,*c*, *qNDPA07a*,* b*,*c*, *q100PWA07a*,* b*,*c* and *q100SWA07a*,* b*,*c* (Fig. S2). Interestingly, the early-maturation allele of this QTL was derived from the late-maturing Hanoch parent that has lower PPP and higher 100PW and 100SW values than IGC99. Moreover, in this locus, Hanoch is also donating higher PPP and lower 100PW/100SW which are opposite to the situation between the parentals. NDP is the only trait in this QTL that is originally higher in Hanoch and is being donated by Hanoch. Therefore, we speculate that *qMIA07a*,* b*,*c* may have originated from both the higher ratio of NDP (positively correlated with MI) and the reduced pod/seed size (negatively correlated with MI). The combination of these two traits may contribute to higher MI in this *qMIA07a*,* b*,*c*.

Analysis of *MIA07* gene list revealed significantly enriched processes related to pod and seed size. For example, the glucan and cellulose biosynthesis pathways, which were enriched in *MIA07*, are known to contribute to structural integrity and flexibility, influencing pod/seed elongation and size in peanut [46] as well as in other crops [46]. A study on peanut pod size mutants found that defects in cellulose synthase genes, along with disruption in plant hormones metabolism, resulted in smaller pod size due to reduced cell elongation [47]. While biological processes related to structural development, energy metabolism, hormonal regulation, and stress adaptation may influence both the growth and developmental timeline of peanut pods, we found no direct evidence linking these processes to plant maturity in peanut. This underscores the need for further molecular studies to eludidate the genetic mechanisms soverning maturity processes in peanut.

Negative correlation of MI to traits such as 100SW and 100PW was previously reported for peanuts [13]. This association can be explained by the fact that seed development requires sufficient time in plants with larger pod size. The correlation between MI and 100SW can also result from the delayed harvest time in late maturing lines, though, as reported [48–50], due to the indeterminate growth habit of peanut [51]. In our case, this is a less likely explanation since the populations were phenotyped in the middle of the growing season (at the midterm of MI between the two parental lines). Interestingly, while QTLs for seed and pod size were found in one of our former *hypogaea* x *hypogaea* populations [8], they did not correlate or co-localize with TTM. We speculate that the large seed/pod size gap between the parental lines dictates whether this trait can directly affect MI. The correlations of MI with SP and PPP and their co-localization are in agreement with previously reported studies [46–48], strengthening the hypothesis that a higher number of pods per plant and rapid pod filling processes may control the reproductive-to-vegetative ratio and promote maturation.

*qMIA07a*,* b*,*c* was also co-localized with *qNDPA07a*,* b*,*c*. As a typical Virginia X Valencia cross, this population is widely segregated to the number of seeds per pod (ranging between 1 and 5). It is obvious that a lower number of seeds per pod (together with the smaller seed size) will promote maturity. The connection between MI and the number of seeds per pod was observed in the other two stable MI loci, *qMIA02a*,* b*,*c* and *qMIA05a*,* b*,*c* co-localized with *qNDPA02a*,* b*,*c* and *qNMPA05a*,* b*,*c*, respectively. Notably, Hanoch is the parental line that donated both early maturity and a higher number of double-seeded pods in *qNDPA02a*,* b*,*c*, while IGC99 is the parental line that donated a higher number of multiple-seeded pods in *qNMPA05a*,* b*,*c*. This result demonstrates that producing more double-seeded pods may promote maturation, even if derived from the late-maturing parental line. The fourth consistent MI QTL, *qMIA10a*,* b*,*c*, and the semi-consistent MI QTL, *qMIB03a*,* b*, did not co-localize with any seed/pod-related traits. In both, the early maturation allele was derived from IGC99, as expected. The observed correlations indicate that selection for early maturity should consider the trade-offs with pod size and yield, a critical factor for peanut breeding strategies aimed at optimizing both maturity and productivity.

Branch length (BL) was previously shown to be associated with peanut pod maturity in ssp. *fastigiata* [14]. The BL trait was negatively correlated with MI, but surprisingly, no QTL association was found. Interestingly, BL was co-localized with BH [7, 19] and also overlapped with previously reported lateral branch length (LBL) QTL [30]. Therefore, we speculate that BL is more connected to branching habit in this population (as erect lines have shorter lateral branches) and less to MI.

Another significant discovery in this study is the identification of *qMIB02*, a TTM QTL that co-localizes with a flowering pattern QTL on linkage group B02. In a previous study, we found that FP is segregating in 1:1 ratio among the RILs in this population, indicating a single gene effect [17]. Flowering pattern was mapped to a small segment within locus *qMSFB02*, wherein a *Terminal Flowering 1-like* (*AhTFL1*) gene with a 1492 bp deletion was found in the *fastigiata* line, leading to a truncated protein [19]. While this QTL was significant in only one environment, its effect was strongly validated in a near-isogenic background, where a sequential flowering pattern was found to accelerate maturity. This result supports the hypothesis that flowering dynamics influence the reproductive phase duration in peanut, which has been previously reported as a determinant of crop maturity [2]. The association between *qMIB02* and the *AhTFL1* gene, a key regulator of flowering time in legumes, further suggests that variations in flowering genes contribute to the genetic architecture of TTM. Additionally, adjusting planting density appears to mitigate yield reductions associated with the determinate growth habit of the MSF plus genotype (B78), offering a potential agronomic strategy to optimize yield in early-maturing lines.

Despite the progress made in mapping stable TTM QTLs, additional work is needed to refine these loci and identify underlying candidate genes. Fine mapping and functional validation, including gene expression analysis and mutant studies, will be necessary to pinpoint causal genetic elements. While such studies were beyond the scope of the current work, our results lay the foundation for future efforts to develop molecular markers for marker-assisted selection (MAS). Given the complex genetic control of TTM, integrating genomic selection approaches with conventional breeding will be essential for accelerating genetic gains in peanut maturity improvement.

Another interesting outcome of the study was the skewed distribution and transgressive segregation observed in the RIL population for MI and in the other traits. MI was strongly skewed toward the late maturing MI values. Several factors could contribute to these patterns, including epistatic interactions, environmental effects, and genetic variation introduced through recombination. Given that TTM may be controlled by four major genes and the pod-related traits are polygenic, their expression likely involves complex genetic interactions. Some allelic combinations from both parental lines may have non-additive effects, leading to transgressive segregation beyond the parental range. The presence of multiple QTLs for TTM and pod traits in different genomic regions suggests that epistatic interactions could contribute to the observed skewness. Environmental Effects may also contributr for this pattern. The study was conducted across three environments, each influencing trait expression differently. G×E interactions may have amplified variation in some traits, particularly in growth-related traits like TTM, where environmental factors such as temperature and soil conditions play a role. The identification of environment-specific QTLs also supports the role of environmental variation in shaping the distribution of traits. However, the same pattern of skewness was found in all three envronments, excluding this factor. Another possible explanation can be sampling bias and segregation distortion. The genetic diversity between ssp. *hypogaea* and ssp. *fastigiata* may have resulted in uneven segregation patterns. The presence of segregation distortion markers in some linkage groups suggests that certain alleles may have been preferentially inherited, contributing to trait skewness. Future studies incorporating fine mapping and epistasis analysis could further clarify the genetic basis of these patterns.

## Conclusions

This study provides new insights into the genetic control of TTM in peanut, emphasizing the interplay between maturity and pod-related traits. The identification of stable QTLs and their co-localization with yield-related loci highlights the importance of a holistic breeding approach that balances early maturation with optimal pod development. This is particularly relevant for Virginia-type peanut breeding, where seed/pod size is a critical trait. Conversely, in cases where seed/pod size is not a major breeding constraint, selecting for early or late maturation can be achieved indirectly by targeting these component traits. Additionally, QTLs not associated with seed/pod size could be valuable for developing early maturing peanut varieties through marker-assisted selection (MAS). These findings contribute valuable genetic resources for peanut improvement programs aimed at developing high-yielding, early-maturing cultivars adaptable to diverse agroecological conditions.

## Methods

### Plant materials

A RIL population was developed from a cross between cv. ‘Hanoch’ and cv. ‘Congo-Red’ (IGC99), as previously described [19]. Hanoch (*A. hypogaea* ssp. *hypogaea* var. *hypogaea*) is a late-maturing (160–170 days from sowing to digging) Virginia-type peanut cultivar widely grown in Israel for in-shell production. It has a spreading growth habit, an alternate flowering pattern with no visible flowers on the main stem (MSF-minus), large double-seeded pods and a pink testa. IGC99 (Israeli Groundnut Collection No. 99) is an old Valencia-type (*A.hypogaea* ssp. *fastigiata* var. *fastigiata*) cultivar that was commercially cultivated in Israel during the 1970 s and 1980s. It exhibits erect lateral branches, sequential flowering, and flowers on the main stem (MSF-plus). IGC99 matures earlier (120–130 days from sowing to digging) and produces smaller seeds than Hanoch, with predominantly 3–4 seeded pods and a red testa.

### Field experiments and trait evaluation

A total of 254 RILs were planted across three field experiments (hereafter reffered to as‘environments’) in commercial peanut-growing plots: 2020 (“20s”) - Nir Itzhak, Western Negev, southern Israel (31°14’15"N34°21’27"E). 2021 (“21s”) - Urim, Western Negev, southern Israel (31°20’17.2"N 34°30’29.2"E). 2021 (“21c”) - Ein HaHoresh, Sharon region, centeral Israel (32°23′09″N 34°56′23″E). The Western Negev locations feature fine sandy-loam soil, while the Sharon site has heavy black soil. These regions also differ significantly in environmental conditions: the Western Negev, located in a desert steppe, has low humidity, whereas central Israel experiences a semi-arid climate with rainy winters and humid summers. RILs were arranged in a complete randomized block design with three replicates. Each genotype was planed in a double row, with beds 4 m in length. spaced 90 cm apart, plants positioned 40 cm apart within each row (total of 20 plants per plot). Parental genotypes were randomly distributed as control plots within the experimental design. Fields were maintained under full-irrigation, and all agronomic practices followed the commercial procedures.

TTM was assessed using the maturity index (MI) value, based on a previously developed method [7, 8]. This method is an adaptation of the hull-scrape method [4] with modifications to accomodate large number of samples. For MI determination, 2–3 plants per plot were sampled. The exocarp of all collected pods was removed using a PICO water pressure machine (Idromatic^®^, Italy) set to 14 MPa with a flow rate of 9 L/min. The pressure-washed pods were then classified into five groups based on mesocarp color: white, yellow, orange, brown, or black. The number of pods in each category was recorded, and MI was calculated as the percentage of pods in the brown and black groups. To capture the widest variation in TTM among the RILs and to determine the optimal sampling date, parental lines underwent maturation tests every few days, beginning at 115 days after planting (DAP). Sampling continued until the early-maturing parent, IGC99, reached an average MI of ~ 70%. Consequently, MI for the entire RIL population was evaluated at ~ 120–130 DAP, depending on the specific environmental conditions. Over the three-locations study, a total of 2286 MI measurements were recorded.

In addition to MI, several traits potentially associated with TTM were recorded across all three environments (20s, 21 s and 21c). Flowering pattern (FP) determined as the presence (MSF-plus) or absence (MSF-minus) of flowers on the mainstem. Branching rate (BR), the number of lateral branches per plant (categorized as ‘up to 5 laterals’ or ‘many laterals’). Growth habit (represented here as Branching Habit; BH), was classified as spreading, bunch or erect. Pod hardness (PH) was classified as hard, medium, or soft based on the manual breaking pressure, using the parental hardness as a reference standard.Pod and seed traits were recorded as well: Number of pods per plant (PPP), number of single-seeded pods (NSP), number of doubled seeded pods (NDP), number of multiple (> 2) seeded pods (NMP), 100 pod weight (100PW), 100 seed weight (100SW) and shelling percentage (SP). Other traits included branch length (BL; cm) that was measured but only in 20 s, and testa color (TC; pink or red). Minimal variations were observed for MSF, BR, BH, PH and TC across environments. Therefore, a single representative value was used for analysis. BH, MSF, BR and BL were recorded at approximately 70 DAP, while PPP was estimated on the same samples used for MI measurement. All remaining pod and seed-related traits were assessed post-harvest.

### Statistical analysis

Parental differences were assessed by Student’s t-test. For the RILs, Shapiro-Wilk test was used to evaluate the normality of trait distributions. If distributions were non-normal, data were transformed (logarithmic or square-root) and reanalyzed for normality. An ANOVA model was applied, including the effects of RIL, Environment, Environment X RIL and Block [Environment]. All effects were treated as random to estimate broad sense heritability (*H*^2^), calculated using the equation $\mathrm H^2=\left({\mathrm\sigma}_{\mathrm g}{}^2/{\mathrm\sigma}_{\mathrm g}{}^2+{\mathrm\sigma}_{\mathrm{ge}}{}^2+{\mathrm\sigma}_{\mathrm e}{}^2{}\right)$, where ${\mathrm\sigma}_{\mathrm g}{}^2{}$, ${\mathrm\sigma}_{\mathrm e}{}^2{}$ and ${\mathrm\sigma}_{\mathrm{ge}}{}^2{}$ denoted the variances of genotypes (G), environment (E) and interaction of genotypes and environments (G x E), respectively. ANOVA and heritability calculations were conducted using QTL IciMapping v4.2.53 (http://www.isbreeding.net/software/?type=detail&id=29). Pearson correlation coefficients were calculated among all traits across the three environments. One-way ANOVA was performed to assess the effects of MSF, BR and BH phenotype on MI. Statistical visualizations, including distribution analysis, histograms, one-way ANOVA and boxplots, were generated using JMP^®^ Pro 17 (SAS Institute Inc., Cary, NC, 1989–2022).

Genetic segregation analysis was conducted using the mixed major gene plus polygene inheritance models [52] implemented by the SEA v2.0 package for R. The 24 candidate models are classified into five groups: (A) one pair of major genes, (B) two pairs of major genes, (C) polygenes only, (D) one pair of major genes plus polygenes, and (E) two pairs of major genes plus polygenes. Distribution parameters for the maturity index in each environment were estimated using the Iterated Expectation and Conditional Maximization (IECM) algorithm. The best-fitting genetic model was selected based on Akaike’s Information Criterion (AIC), likelihood ratio tests, and a series of goodness-of-fit statistics.

The SNP genotyping and genetic map construction procedures were previously described in Kunta et al., 2022a [19] and are summarized here. SNP genotyping was performed using the Affymetrix Axiom_*Arachis*2 SNP-array, which includes 47,837 SNPs [53, 54]. Axiom Analysis Suite 3.1 was used for genotypic data processing, as previously described [55]. Briefly, polymorphic homozygous SNPs (AA and BB) and polymorphic heterozygous SNPs (AA or BB and AB) with 65 − 35% call-rate frequencies were retained among the RILs. SNP Marker data with more than 10% missing data and more than 20% heterozygote calls (in AA/BB parental SNPs) were removed [56]. The final SNP dataset, with minor adjustments for 254 RILs, was used to construct the genetic linkage map in Joinmap v4.1 [57], using a minimum LOD of 3.0 and the Haldane mapping function. Graphical representations of the linkage maps were generated in MapChart v2.3 [58]. Loci positions were validated as described by Chavarro et al., 2020 [56], with minor modifications (BLASTN (e-value < 1 × 10^− 18^) and mismatch of less than 2). The resulting genetic linkage groups (LGs) were assigned to the pseudo-molecules of *A. hypogaea* cv. Tifrunner.

### QTL mapping

Mapping of MI, PPP, NSP, NDP, NMP, 100PW, 100SW, SP, PH, TC and BL was performed on the 254 RILs using MapQTL v6 [59]. PH, and TC trait mapping was done by converting each qualitative phenotype into a numerical value. Interval mapping was conducted using the maximum likelihood algorithm with a LOD score of 2.5, determinated through 1000 permutations at a 95% significance level. QTLs with explaining more than 10% of the phenotypic variation (PVE) were defined as major QTLs, while those explaining less than 10% PVE were classified as minor QTLs [60]. Previously reported QTL mapping results for MSF, BR and BH [19] were used to assess the genomic association of these traits with MI. The QTL naming followed the standard terminology: “*q*” denotes a QTL, followed by an abbreviation of the trait. The last digit indicates the linkage group (LG), and if the QTL was identified in multiple environments, alphabetical order is used (a = 20 s, b = 21 s, c = 21c). If multiple QTLs were found on the same LG, they are numbered sequentially. For example, *qMIA02a*,-*b*,-*c* and *qMIB03a*,-*b* represent QTL identified in three environments and two environments, respectively.

### Gene ontology analysis

Gene ontology (GO) annotations for all protein coding genes were downloaded from the Legume information system database (https://mines.legumeinfo.org/arachismine/begin.do). Enrichment analysis was generated with Blast2Go [version 5.2.5] using Fisher’s exact test. The test was performed for each of the two gene sets separately, the GO annotations of all *A. hypogaea* [genome build 1] protein coding genes served as background vs. GO annotations of proteins within the *qMIA07* QTL. GO annotation with p-value < 0.05 was considered significant.

### Validation of the *qMIB02* effect in Hanoch genetic background

A field trial was conducted to assess the effect of *qMIB02* on maturity level within the Hanoch genetic background. Two genotypes were compared, Hanoch and line B78. Line B78 originated from EMS line 212, a Hanoch-based mutant line exhibiting a sequential flowering pattern [19]. Line 212 was crossed with Hanoch, followed by two additional backcrosses, while maintaining the sequential flowering pattern. B78 was selected from a BC_2_F_3_ family that exhibit a stable sequential flowering pattern. The trial was conducted in Magen, Israel, in a commercial field plot (commercial variety: Hanoch). Three treatments were performed: Hanoch with three rows per bed, B78 with three rows per bed, and B78 with five rows per bed. The five-row treatment aimed to test whether increasing planting density could compensate for the decrease in yield line B78 due to its determinate nature. The experimental design was randomized block design with eight replications. In each replication and treatment, plots of 6 m bed were sown at a commercial stand density (five plants per meter), using mechanized sowing with manual completion in the five-row-per-bed treatment. Maturity tests were performed at three intervals: 127, 141 and 157 days from sowing, following the same methodology described earlier for the RIL population. At the end of the season, after uprooting, the crop was harvested manually, and an estimate of the pod yield per squared meter was recorded.

## Supplementary Information

Supplementary Material 1.

Supplementary Material 2.

Supplementary Material 3.

Supplementary Material 4.

Supplementary Material 5.

Supplementary Material 6.

## Data Availability

All the data is directly available from the main manuscript and the supplementary files.

## Declarations

### Supplementary tables legends

Table **S1**. Phenotypic trait values of MI and the component traits in 254 Hanoch X IGC99 RILs.

Table **S2.** Comparison of 24 segregation models for maturity index in peanut, ranked by Akaike’s Information Criterion (AIC). Each value is an average of the three replications (environments).

Table **S3.** Hanoch X IGC99 RIL population, linkage map order with corresponding physical positions. The physical position is based on the tetraploid peanut sequence [42].
