# Supplementary figures and images for: Genetic mapping of time-to-maturity trait in hypogaea x fastigiata peanut background reveals a significant effect of pod-related and flowering pattern
Source: BMC Plant Biol. 2025 Dec 1;26:27. doi: 10.1186/s12870-025-07707-z (PMC12777413; doi:10.1186/s12870-025-07707-z)

**Fig. S1.**

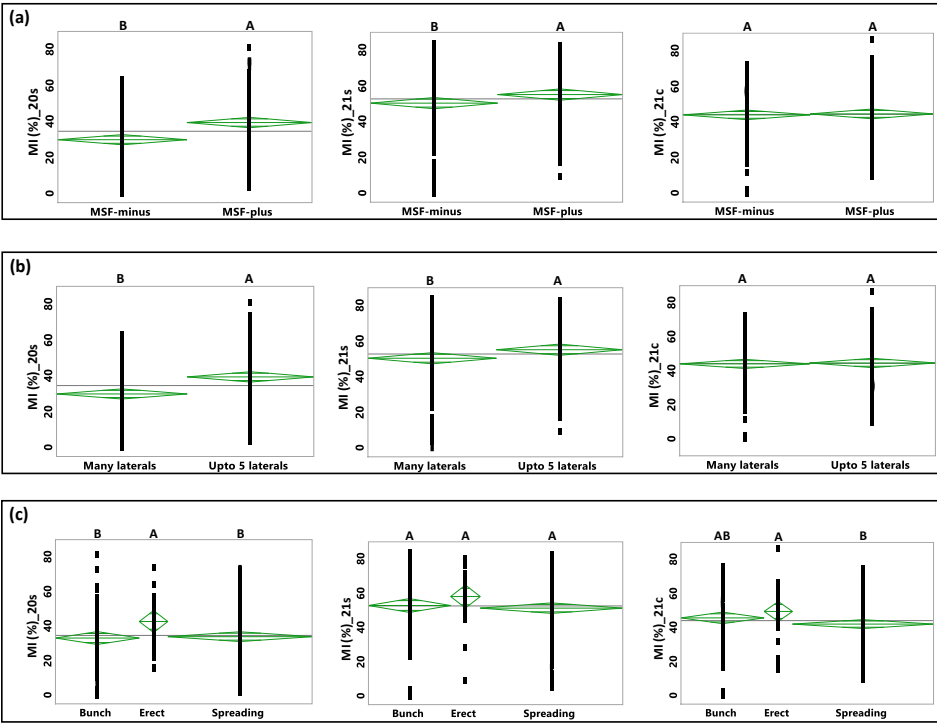

Supplement: Supplementary file 4 — Supplementary Material 4. [file 12870_2025_7707_MOESM4_ESM.pdf]

Fig. S3.

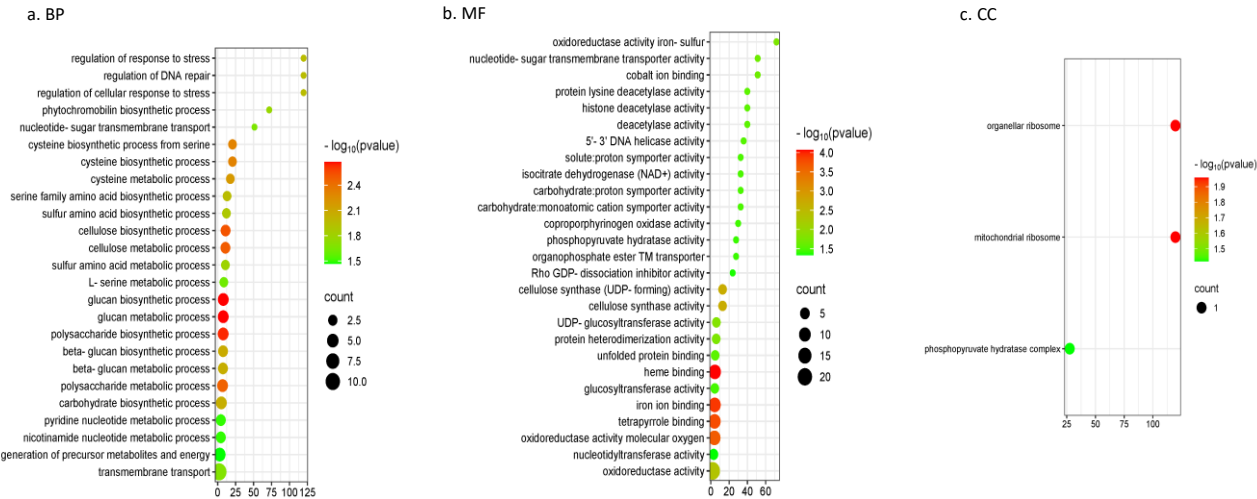

Supplement: Supplementary file 5 — Supplementary Material 5. [file 12870_2025_7707_MOESM5_ESM.pdf]

**Fig. S2.**

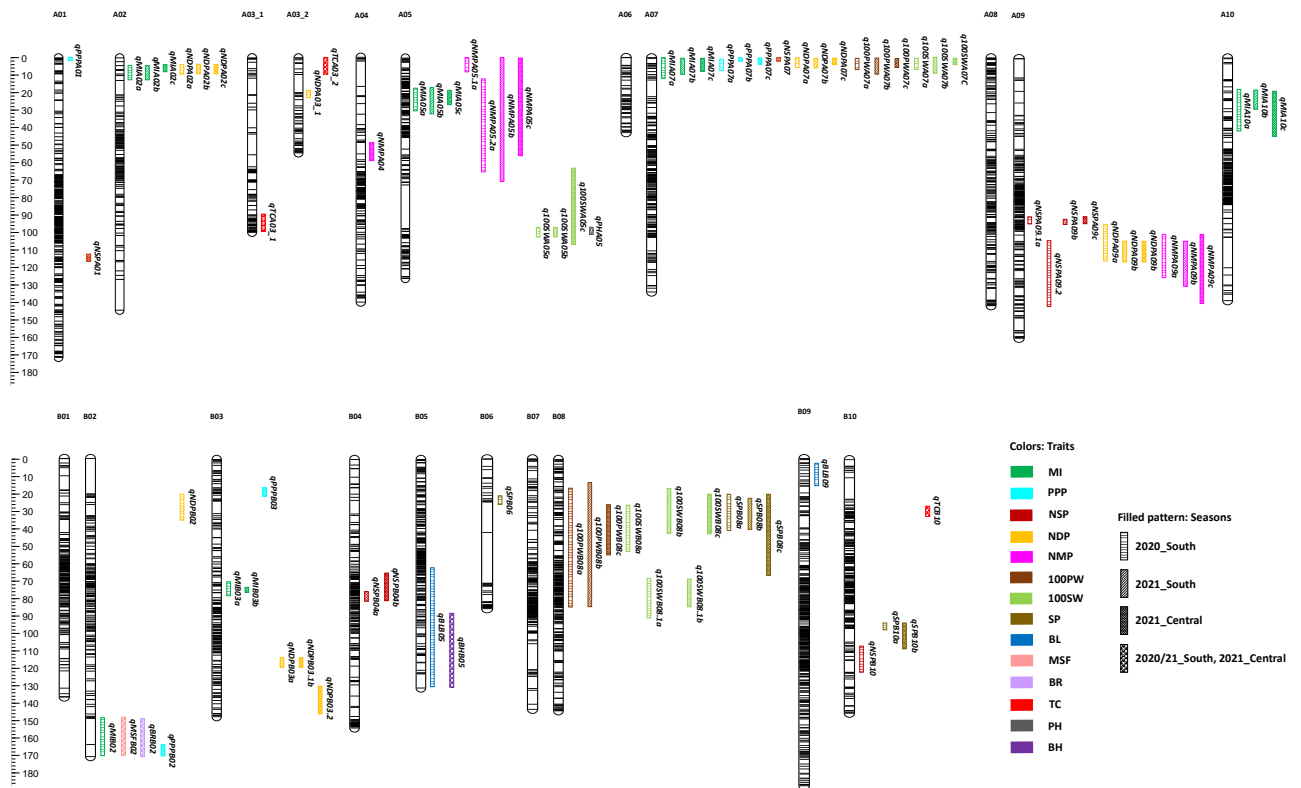

Supplement: Supplementary file 6 — Supplementary Material 6. [file 12870_2025_7707_MOESM6_ESM.pdf]
